# The Law Relating to Lunatics and Persons of Unsound Mind.—I

**Published:** 1903-01-31

**Authors:** J. E. R. Stephens

**Affiliations:** Barrister-at-Law, Author of "Digest of Public Health Cases"


### THE LAW RELATING TO LUNATICS AND PERSONS OF UNSOUND MIND.—I.

By J. E. R. Stephens, Barrister-at-Law, Author of "Digest of Public Health Cases."

Every person whose mind from his birth by a perpetual infirmity is so deficient as to be incapable of directing him in any matter which requires thought or judgment, is in legal phraseology *an idiot.* Every person *qui gaudet lucidis intervallis,* and who sometimes is of good and sound memory, and sometimes *non compos mentis,* is in legal phraseology *a lunatic.* Every person who, by reason of a morbid condition of intellect, is as incapable of managing himself and his affairs as an idiot or a lunatic, not being an idiot or lunatic, or a person of merely weak mind, is in legal phraseology *a person of unsound mind.* It must be remembered, however, that in legal phraseology a person whose moral feelings are perverted is not by reason of such perversion a person of unsound mind. Further, that if the mind is unsound on one subject, it is not sound on any subject, the mind being indivisible. *Non compos mentis* is the legal generic term which includes the three several classes just mentioned.

A lucid interval is "a perfect restoration to reason"—"a suspension, not a mere remission, of the morbid state." The restoration of a mind to its original strength is not, however, necessary to establish a lucid interval. The proof of a lucid interval is extremely difficult, particularly in the case of habitual insanity; and the law requires very clear proof of its existence, such proof, indeed, must be "as clear and demonstrative as the legal proof of insanity supervening on previously uninterrupted sanity." All the circumstances of an act in an alleged lucid interval should be evidenced; an independent rational act done in a rational manner contributes materially to the proof of a lucid interval; on the other hand, the nature of an act may be some evidence of its not having been done during a lucid interval.

In a medico-legal view the existence of a lucid interval must be always looked upon with suspicion and doubt when the interval alleged is very short, and "the probabilities *a priori* in favour of a lucid interval are stronger in a case of delirium than in one of proper permanent insanity." The rational rule has been laid down that persons in the habit of watching a lunatic can best prove his lucid intervals. A lucid interval having been once proved, all acts done during its existence are of course valid.

From predilection for liberty, and because the state of mind of the idiot, the lunatic, and the person of unsound mind is abnormal, the presumption of English law is in favour of sanity, and this presumption even extends to the case of a person born deaf and dumb, or deaf, dumb, and blind, although in the payment of money to a person born deaf, dumb, and blind a court of justice will exercise much caution. If, however, this presumption in favour of sanity is once rebutted by proof of habitual insanity, the law presumes no lucid interval or restoration to sanity; such interval or restoration must always be established by evidence.

An idiot may in general be clearly identified "by a peculiar physiognomy, an absence of all expression, and a vague and unmeaning look;" or by "the endless repetition of short sentences, or portions of sentences." In fact "there are many ways in which idiocy in manifested, as by neglect of the ordinary decencies of life, frequently by foolish laughter, by general vacancy of aspect, and by other modes which are better understood when seen than by any verbal description. There is also a test from an incapacity of understanding numbers;" but any "acts of business disprove idiocy."

A lunatic, or person of unsound mind, is not so easily identified as an idiot; the legal test of a lunatic or unsound mind, where there is no frenzy, is the existence in the mind of a fixed extravagant delusion. Sir J. Nicholls, in *Daw* v. *Clark* (5 Russ. 168), adopted as a legal definition of the delusion which haunts the lunatic or unsound mind, "a belief of facts which no rational person would have believed," but Lord Brougham, in *Waring* v. *Waring* (6 Moore P.C., 349–354), that a more accurate definition would be "the belief of things as realities which exist only in the imagination of the patient." Dr. Haslam has also declared that "false belief is the essence of insanity," and his opinion is confirmed by Dr. Mayo, who says that "the state of mind of the lunatic or person of unsound mind is marked by delusion or by inconsecutive and incoherent trains of thought, generally by both conditions," and by Dr. Battie, who thus expresses himself: "Deluded imagination is not only an indisputable, but an essential character of madness."

"Great general eccentricity assists materially in the proof of lunacy or unsoundness of mind," and "furnishes strong ground for suspicion of predisposition to madness"; but prolonged departure, without an adequate external cause, from the habits of feeling and thinking exhibited by an individual in the previous tenor of his life, distinguishes the lunatic or person of unsound mind from the merely eccentric man. Indeed, "change of character is the great feature of insanity." The language of Lord Langdale, Master of the Rolls, is also to this effect: In *Snook* v. *Watts* (11 Beav. 107) he observes, "A man may be subject to delusions, and one of the means, and perhaps the most accurate means, of judging whether these apparent indications ought to be relied upon as proving a general unsoundness of mind, is by a comparison of the alleged acts of insanity with other acts of the same person and the general course of his life. When a man's ways and general course of life are such as to indicate sanity and a knowledge of his affairs, proof of one or more particular acts, though very strange in themselves, and though affording some grounds for imputing insanity, would not be a sufficient proof to show that all his acts were done under the delusion of insanity. On the other hand, when a man is thought by various persons to have been insane at a particular period, and to have so continued ever since, proof of one or more acts done afterwards, apparently in the manner of a man of unsound mind, would not, if unaccompanied by other proof and the application of some tests or inquiry prove that the acts done were done under circumstances free from deluson, or, what is quite as much of importance, free from the influence to which persons acting under insane delusions are confessedly liable." Rational acts are separately no proof that a person is not a lunatic or of unsound mind when the feature of his mental disorder is not frenzy, but a fixed extravagant delusion, because the delusion may not be called forth or may be studiously concealed.

Proof of insanity must be evidenced by facts, and "where insanity has not previously existed, proof of insanity is not to be made out by rambling through the whole life of a party, but must be applied to the particular date of any act impeached, on the ground of his insanity." Lord Chancellor Eldon said that it was not a proper mode of proceeding in a question of sanity merely to state facts to medical men and take their opinion upon these facts without any previous personal examination by them of the person whose sanity is in question.

It is "a principle of natural justice and of our law, that, *actus non facit reum nisi mens sit rea*, the intent and the act must both concur to constitute crime." Now, intent (which has been defined to be "the design, purpose, or fixed direction of the mind to some particular object") implies mental capacity. Therefore, it seems, if a person *non compos mentis* has sufficient mental capacity fully to understand the nature of any act when committing it in violation of the criminal law, he is in respect of such act punishable as a criminal by that law; if he has not that degree of mental capacity, he is irresponsible criminally in respect of such act. The following questions were, in regard to *McNaghten's case* (8 Scott, N. R. 600), proposed to the judges by the House of Lords, and the following answers were returned by them:—

Question I. What is the law respecting alleged crimes committed by persons afflicted with insane delusions in respect of one or more particular subjects or persons, as, for instance, when at the time of the commission of the alleged crime the accused knew he was acting contrary to law, but did the act complained of with a view, under the influence of insane delusion, of redressing or revenging some supposed grievance or injury, or of producing some supposed public benefit?

Question II. What are the proper questions to be submitted to the jury when a person alleged to be afflicted with insane delusions respecting one or more particular subjects or persons is charged with the commission of a crime (murder for example) and insanity is set up as a defence?

Question III. In what terms ought the question to be left to the jury as to the prisoner's state of mind at the time when the act was committed?

Question IV. If a person under an insane delusion as to existing facts commits an offence in consequence thereof, is he thereby excused?

Question V. Can a medical man conversant with the disease of insanity, who never saw the prisoner previously to the trial, but who was present during the whole trial and the examination of all the witnesses, be asked his opinion as to the state of the prisoner's mind at the time of the commission of the alleged crime, or his opinion whether the prisoner was conscious at the time of doing the act that he was acting contrary to law, or whether he was labouring under any and what delusion at the time?

The joint answers of all the judges, with the exception of Mr. Justice Maule, who gave a separate answer, were as follows:—

Answer of the judges to Question I. Assuming that the inquiries are confined to those persons who labour under such partial delusions only, and are not in any other respects insane, we are of opinion that, notwithstanding the party accused did the act complained of with a view, under the influence of insane delusion, of redressing or revenging some supposed grievance or injury, or of producing some public benefit, he is nevertheless punishable according to the nature of the crime committed if he knew at the time of committing such crime that he was acting contrary to law.

As Questions II. and III. appear to us to be more conveniently answered together, we have to submit our opinion to be that the jury ought to be told in all cases that every man is presumed to be sane and to possess a sufficient degree of reason to be responsible for his crimes until the contrary be proved to their satisfaction; and that to establish a defence on the ground of insanity, it must be clearly proved that at the time of committing the act the party accused was labouring under such a defect of reason from disease of the mind as not to know the nature and quality of the act he was doing; or, if he did know it, that he did not know he was doing what was wrong. The mode of putting the latter part of the question to the jury on these occasions has generally been whether the accused at the time of the doing of the act knew the difference between right and wrong, which mode, though rarely, if ever, leading to any mistakes with the jury, is not, as we conceive, so accurate when put generally, and in the abstract, as when put with reference to the party's knowledge of right and wrong in respect to the very act with which he is charged. If the question were to be put as to the knowledge of the accused solely and exclusively with reference to the law of the land, it might tend to confound the jury by inducing them to believe that an actual knowledge of the law of the land was essential in order to lead to a conviction, whereas the law is administered upon the principle that everyone must be taken conclusively to know it, without proof that he does not know it. If the accused was conscious that the act was one which he ought not to do, and if that act was at the same time contrary to the law of the land, he is punishable; and the usual course therefore has been to leave the question to the jury, whether the party accused had a sufficient degree of reason to know that he was doing an act that was wrong, and this course we think is correct accompanied with such observations and explanations as the circumstances of each particular case may require.

The answer to question IV. must of course depend on the nature of the delusion; but making the same assumption as we did before, namely, that he labours under such partial delusion only, and is not in other respects insane, we think he must be considered in the same situation as to responsibility as if the facts with respect to which the delusion exists were real. For example, if under the influence of his delusion, he supposes another man to be in the act of attempting to take his life, and he kills that man as he supposes in self defence, he would be exempt from punishment. If his delusion was, that the deceased had inflicted a serious injury to his character and fortune, and he killed him in revenge for such supposed injury, he would be liable to punishment.

In answer to question V., we think the medical man under the circumstances supposed cannot in strictness be asked his opinion in the terms above stated, because each of these questions involves the determination of the truth of the facts deposed to, which it is for the jury to decide, and the questions are not mere questions of a matter of service, in which case such evidence is admissible. But where the facts are admitted or not disputed, and the question becomes substantially one of science only, it may be convenient to allow the question to be put in that general form, though the same cannot be insisted upon as a matter of right.

